# Population Structure as Revealed by mtDNA and Microsatellites in Northern Fur Seals, *Callorhinus ursinus*, throughout Their Range

**DOI:** 10.1371/journal.pone.0010671

**Published:** 2010-05-17

**Authors:** Bobette R. Dickerson, Rolf R. Ream, Sacha N. Vignieri, Paul Bentzen

**Affiliations:** 1 Molecular Ecology Research Laboratory, National Marine Mammal Laboratory, Alaska Fisheries Science Center, National Marine Fisheries Service, Seattle, Washington, United States of America; 2 Department of Organismic and Evolutionary Biology, Museum of Comparative Zoology, Harvard University, Cambridge, Massachusetts, United States of America; 3 Dalhousie University, Halifax, Nova Scotia, Canada; University of Otago, New Zealand

## Abstract

**Background:**

The northern fur seal (*Callorhinus ursinus*; NFS) is a widely distributed pinniped that has been shown to exhibit a high degree of philopatry to islands, breeding areas on an island, and even to specific segments of breeding areas. This level of philopatry could conceivably lead to highly genetically divergent populations. However, northern fur seals have the potential for dispersal across large distances and have experienced repeated rapid population expansions following glacial retreat and the more recent cessation of intensive harvest pressure.

**Methodology/Principal Findings:**

Using microsatellite and mitochondrial loci, we examined population structure in NFS throughout their range. We found only weak population genetic structure among breeding islands including significant F_ST_ and Φ_ST_ values between eastern and western Pacific islands.

**Conclusions:**

We conclude that insufficient time since rapid population expansion events (both post glacial and following the cessation of intense harvest pressure) mixed with low levels of contemporary migration have resulted in an absence of genetic structure across the entire northern fur seal range.

## Introduction

**Figure 1:**
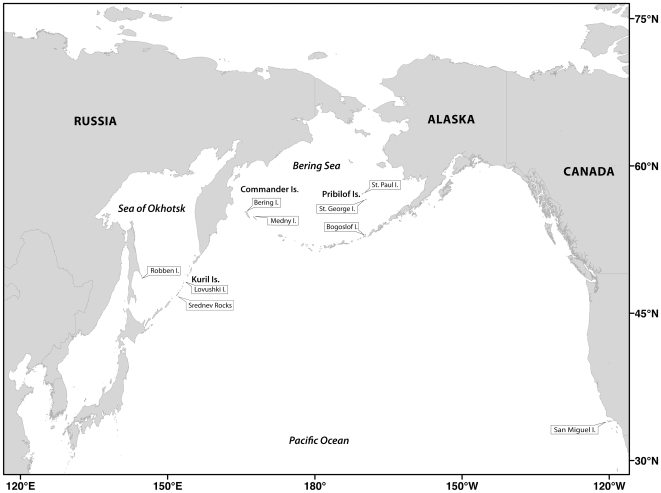
Distribution of northern fur seal breeding sites.

The northern fur seal (*Callorhinus ursinus*) is a widely distributed member of the family Otariidae with a pelagic distribution across the North Pacific Ocean from the Sea of Okhotsk to the northern Bering Sea and as far south as 34° N [1], [2]. Breeding among this species occurs on a limited number of islands within this range: Robben Island, the Kuril Islands (Lovushki and Srednev), and the Commander Islands (Bering and Medny) in Russia; Bogoslof Island and the Pribilof Islands (St. George and St. Paul) in Alaska; and San Miguel Island in California (Figure 1). Most of these islands contain several distinct breeding areas. Individuals of this long-lived species exhibit a predictable annual pattern of seasonal pelagic migration from the islands into the North Pacific in late fall, returning to breed and rear young in late spring and throughout the summer [2].

Northern fur seals have a highly polygynous mating system, and both sexes exhibit philopatry to islands, breeding areas on an island, and even to specific segments of breeding areas [1]–[6]. Baker *et al.* [6] examined harvest data and found that, for females that were at the average age of first reproduction, 84% were killed at their natal breeding area or adjacent haulout within an island. Further, in a set of data that did not include females killed on adjacent haulouts, the homing rate was 92% or greater for all age classes. These rates may still be underestimates because of the propensity of females to make brief visits to breeding areas other than their parturition site [2]; there were no data indicating that the females had pups at the harvest site. Baker *et al*. [6] also examined tag-resight data for juvenile male fur seals and found that, for 5-year-olds, 73%–84% were at their natal breeding area within an island when first recaptured. These rates are probably underestimates, as well. For juvenile males recaptured more than once within a summer, the likelihood of observing an animal at its natal breeding area within an island increased significantly with time between recaptures. Eleven days or more after the first recapture, 100% of 5-year-old juvenile males were found at their natal breeding area within an island. The precision of philopatry can also be remarkable in northern fur seals. Gentry [2] observed individual females that showed fidelity to the same territory of their own birth, and was able to estimate that they return to produce offspring an average of 8.3 m from their natal territory. Chelnokov [5] reported similar observations for territorial male northern fur seals; of 14 males resighted at their natal breeding area, 13 held territories on the section where they had been born. However, as would be expected in a wide-ranging pelagic species, movement among islands does occur, as evidenced by the colonization and rapid growth on Bogoslof Island in 1980 [7], [8], and San Miguel Island in 1965 [9]. Further evidence of the capacity for northern fur seals to move large distances is supported by a number of telemetry studies that show that females can travel ∼200 km [10], [11] and juvenile males ∼400 km [12] from their rookery on foraging trips during the breeding season. Further, during their winter migrations both sexes travel distances (thousands of km) large enough to encompass multiple breeding colonies [13], [14]. These types of long distance migrations generally occur outside of the breeding season or by sexually immature animals [9] with breeding site fidelity increasing with the onset of sexual maturity [6].

Species that display a high degree of philopatry might be expected to exhibit significant genetic differentiation between breeding colonies due to reproductive isolation. Movement of animals between breeding colonies, on the other hand, would reduce the differences seen between populations and even small numbers of migrations can result in population homogenization. Steller sea lions, *Eumetopias jubatus*, occupy an overlapping range with northern fur seals and have at least the same dispersal capacity but are found to have genetic differentiation suggesting the presence of several stocks [15], [16]. Further complicating an investigation into northern fur seal population dynamics is the fact that they have undergone a number of population expansion and decline events. Much of their current geographic distribution was unavailable until ∼10,000 ybp due to the extensive ice sheets of the Wisconsin glaciation (last glacial maximum 18,000–20,000 ybp) [17]. More recently, commercial harvests contributed to large reductions and fluctuations in northern fur seal abundance during the past 200 years [2], [18], [19]. Recolonization following these perturbations could have a substantial homogenizing influence on their current genetic patterns. Rapid recolonization following glacial retreat and cessation of hunting pressures has been seen in other otarids including; Cape fur seal, *Arctocephalus pusillus pusillus* [20], hooded seal, *Cystophora cristata* [21], Juan Fernandez fur seal, *A. philippii*, [22], New Zeland fur seal, *A. forsteri* [23] and Antartic, and subantarctic fur seal, *A. gazelle* and *A. tropicalis* [24]. In some cases the resulting populations showed at least moderate genetic differentiation between colonies [22]–[24] and in others the breeding population appears to be panmictic [20], [21].

The current size of the northern fur seal population is about 1.2 million individuals, of which ∼50% are found on the Pribilof Islands (National Marine Fisheries Service unpublished data). Pup production on the Pribilof Islands has declined precipitously over the past decade, however, and the cause has yet to be identified [25]. This decline was preceded by substantial declines during 1956–1980 that were attributed to an experimental harvest of females [26]. The Pribilof Island population has now reached a level as low as those observed during the early 1900s, when the population was recovering from vast unregulated pelagic harvests. To date, there have been no molecular studies of genetic variation or population structure in the northern fur seal. In this study we examine range-wide population structure at mitochondrial and microsatellite markers in northern fur seals.

## Methods

### Ethics Statement

The Marine Mammal Commission in Washington DC approved the protocol for sample collection and all samples were collected in accordance with the Marine Mammal Protection Act guidelines, under the authority of Permit Numbers 837 and 782–1708.

### Sample collection and preparation

Small pieces of skin were collected from the front or hind flipper of northern fur seal pups and stored in 100% ethanol at room temperature. During the 1993 to 1998 summer breeding seasons, skin samples (n = 578) were obtained from eight islands on which fur seals breed: Bering Island (55), Bogoslof Island (99), Lovushki Island (11), Medny Island (56), Robben Island (50), San Miguel Island (94), St. George Island (100), and St. Paul Island (113) (Figure 1) for use in both the microsatellite and mtDNA analyses. Genomic DNA was extracted from these samples following a standard phenol:chloroform method [27] and resuspended in TE buffer (10 mM Tris, 0.1 mM EDTA, pH 8.3). During the 2005 breeding season additional samples were collected from the Kuril Islands (Lovushki; 50 and Srednev; 50) and San Miguel Island (50) for use in the mtDNA analysis. Genomic DNA was isolated from these samples using DNeasy® tissue kits (Qiagen, Valencia, CA).

### Microsatellite amplification

Seven loci, Hg3.7 [28], Hg4.2, Hg6.3 and Hg8.10 [29], M2b [30], M11a [31] and SPGv11 [32], were selected based on length, annealing temperature, and quality of allele amplification. Polymerase chain reactions (PCR) were performed on a Perkin Elmer 9600 thermocycler in 10 µL volumes (10 mM Tris-HCl, 50 mM KCl, 2.0 mM MgCl_2_, 0.2 mM each dNTP, 0.3 U *Taq* DNA polymerase, and 100 ng DNA template). PCR profiles consisted of one cycle at 94 °C for 2 min; 5 cycles of 94 °C for 30 s, *x*+5 °C, decreasing 1 °C each cycle (touchdown PCR), for 30 s, and 72 °C for 15 s; 23 cycles of 94 °C for 30 s, *x* °C for 30 s, and 72 °C for 15 s; and one cycle at 72 °C for 30 min; where *x* is the annealing temperature of the primer. Following amplification the products were visualized on an ABI 373A automated sequencer (Applied Biosystems, Foster City, CA). Genotypes were scored using GeneScan 672 and Genotyper version 2.0 software (Applied Biosystems, Foster City, CA). To control for scoring errors, on each gel two individuals were rerun and rescored anonymously. Histograms were constructed for each locus, using all individuals, and bins were created to score relative allele size, thus avoiding allele scoring errors caused by adjacent alleles differing in called size from expected values for the repeat length and number of repeats [33]. Alleles were scored for each locus using these bins and tables were created using the Genotyper software.

### Mitochondrial DNA amplification and sequencing

PCR was used to amplify the ∼375 base pair (bp) target sequence from the control region of the mtDNA using primers LGL 283 (5′-TACACTGGTCTTGTAAACC-3′) [34] and PINN 1115 (5′-ATGGCCCTGAAGTAAGAAGAACCAG-3′) (slight modification from LGL 1115 of Bickham et al. [34] for greater specificity). The PCR was conducted in a 10 µL volume consisting of 10 mM Tris-HCL at pH 8.3, 50 mM KCL, 2.0 mM MgCl_2_, 0.8 mM deoxynucleotide triphosphates (dNTPs, 0.2 mM each), 0.1 U *Taq* DNA polymerase, 0.2 µM of each primer, and 100 ng DNA template. PCRs were performed on a MJ Research DNA engine (Waltham, MA) under the following profile: 30 cycles of 93°C for 20 s, 59°C for 20 s, and 72°C for 35 s.

PCR products were visualized via electrophoresis on 1.5% agarose gels at 100 v for 1.5 hours. The gels were stained with SYBR Green I nucleic acid gel stain (Molecular Probes INC) and viewed with a UVP Darkroom (UVP, Upland, CA) with sizes verified with Hi-Lo DNA marker (Minnesota molecular, Minneapolis, MN). To purify the amplified PCR fragment the bands were excised from the gel and placed in 20 ul of low TE buffer (10 mM Tris, 0.1 mM EDTA, pH 8.3) and stored overnight at 4°C.

The subsequent cycle sequence PCRs were performed using the Thermo Sequenase Primer Cycle Sequencing Kit (Amersham Biosciences) protocols in a MJ Research DNA engine (Waltham, MA) using fluorescently labeled primers. Sequences were visualized using a Li-Cor 4200 automated sequencer (Li-Cor Biosciences, Lincoln, NE) and base calling and sequence editing were done with the associated E-seq software (Li-Cor Biosciences, Lincoln, NE). All haplotypes have been submitted to GenBank (accession numbers EU791990–EU792321).

### Analysis

#### Microsatellites

Calculations of observed and expected heterozygosity and tests for Hardy-Weinberg equilibrium (HWE), genotypic disequilibrium, and population differentiation were conducted using GENEPOP version 3.1d [35]. Tests for conformity to HWE were conducted for each locus-population combination, and for genotypic linkage disequilibrium for all pairs of loci within and across populations. An unbiased estimate of the exact *P*-value was determined using a Markov chain method following the permutation algorithm of Guo and Thompson [36]. STRUCTURE version 2.1 [37] was used to estimate the most likely number of populations (K) represented by the entire sample set, using the admixture model with 50,000 steps conducted as “burn-in followed by 100,000 Markov chains, with three iterations per K. We tested for between 1 and 8 K, where 8 would indicate a distinct population for each island. Population differentiation was tested between all population pairs and among all populations, at each locus and over all loci, using FSTAT [38], [39] and GENEPOP to compute unbiased estimates of F_ST_ [40]. We tested if the F_ST_ estimates for all populations, and for population pairs across loci, were significantly greater than zero by permuting multi-locus genotypes among samples with FSTAT. Mantel tests [41] were conducted in GENEPOP to test for isolation by distance, using the natural logarithm of geographic distance and linearized estimates of F_ST_/(1-F_ST_) for each population pair. Allelic richness was calculated using FSTAT (due Lovushki's small sample size (N = 11) it was excluded from this analysis) and an analysis of variance was used to compare the mean allelic richness, across all loci, of populations in the eastern Pacific (Medny Island, Robben Island, and Bering Island) to those in the western Pacific (Bogoslof Island, St. Paul Island, St. George Island, and San Miguel Island).

#### Mitochondrial DNA

Sequencher version 4.7 (Gene Codes, Ann Arbor, MI) was used to align forward and reverse sequences and to create a consensus for each sample. Consensus sequences from all samples were aligned in Bioedit, version 7.0 [42]. Arlequin, version 3.01 [43] was used to determine the number of variable sites, identify haplotypes and to calculate genetic diversity (on both the haplotype and nucleotide level). To investigate population structure among regional population groupings, an analysis of molecular variance (AMOVA) was performed in Arlequin using the following six groups: Robben Island, the Kuril Islands (Lovushki and Srednev), the Commander Islands (Bering and Medny), Bogoslof Island, the Pribilof Islands (St. George and St. Paul) and San Miguel Island. A broad regional comparison between islands in the western Pacific (Robben Island, Kuril Islands, and the Commander Islands) and islands in the eastern Pacific (Pribilof Islands) was also conducted with an AMOVA. This comparison was limited to historical fur seal breeding sites by excluding recently colonized islands (Bogoslof Island and San Miguel Island). Pairwise comparisons of F_ST_ and Φ_ST_ among all islands were conducted in Arlequin.

A test for isolation by distance was performed by regressing Φ_ST_/1- Φ_ST_ against the natural logarithm of geographical distance using GENEPOP, version 3.4 [35]. To investigate the recent demographic history of this species a minimum spanning network was created in Arlequin, version 3.01 [43] and drawn by hand. To test for the signature of rapid expansion, we created a nucleotide mismatch frequency distribution in DnaSP, version 4.10.9 [44] and compared this to a model of sudden population expansion [45], [46].

To estimate past population sizes we used BEAST 1.4.8 [47] to construct a Bayesian skyline plot (employing the Bayesian MCMC coalescent method, a GTR+G+Γ model of substitution [MODELTEST 3.7 [48]], and a strict clock). The Bayesian distribution was generated using 425 million MCMC steps, in blocks of 10 million steps until effective samples sizes (ESS) of parameter estimates exceeded 200. We assumed a generation time of 15 years and a mutation rate of 5.8% per million years. This mutation rate was calibrated in BEAST against 90 pinniped sequences: 32 modern northern fur seals, 30 Stellar sea lions (*Eumetopias jubatus*) and 28 California sea lions (*Zalophus californianus*) assuming a 8.2+/−2.1 mya divergence time between sea lions and northern fur seals [49] and an HKY+I+G mutation model with a strict clock for 20 million steps [47]. This rate fell within the 5–10% per million years estimated for southern elephant seals (*Mirounga leonine*) control region [50].

## Results

Considerable variation was observed at all microsatellite loci. The total number of alleles at each locus ranged from 12 (SGPv11; mean  = 7.4) to 22 (M2b; mean  = 16.8; Table 1). Only locus SGPv11 had an average observed heterozygosity less than 83% (29%; Table 1). There were no significant departures from HWE observed among locus-population combinations indicating that we did not have notable problems with null alleles.

**Table 1:** Summary of allelic variability at seven microsatellite loci in eight populations of northern fur seals.

		Locus							
Population		Hg3.7	Hg4.2	Hg6.3	Hg8.10	M2b	M11a	SGPv11	Mean
Bering	N	49	50	47	47	50	48	47	48.6
	*A*	13	15	10	13	19	15	7	12.8
	*H_O_*	0.694	0.860	0.894	0.915	0.940	0.896	0.234	
	*H_E_*	0.837	0.881	0.864	0.883	0.897	0.909	0.256	
Bogoslof	*N*	99	97	94	96	99	99	95	97.1
	*A*	16	16	11	15	20	15	9	14.4
	*H_O_*	0.889	0.866	0.862	0.875	0.929	0.919	0.221	
	*H_E_*	0.808	0.883	0.870	0.884	0.909	0.902	0.287	
Lovushki	*N*	10	11	10	8	11	11	11	10.3
	*A*	8	10	5	7	9	9	4	7.4
	*H_O_*	0.900	1.000	0.700	0.750	0.909	0.909	0.273	
	*H_E_*	0.847	0.900	0.816	0.875	0.875	0.892	0.403	
Medny	*N*	23	36	28	25	35	25	34	30.3
	*A*	10	13	12	14	16	13	6	11.6
	*H_O_*	0.870	0.889	0.821	0.880	0.829	0.880	0.265	
	*H_E_*	0.839	0.876	0.855	0.907	0.895	0.889	0.295	
Robben	*N*	46	49	48	49	48	48	49	48.0
	*A*	10	14	10	14	18	13	8	12.4
	*H_O_*	0.739	0.980	0.854	0.918	0.875	0.917	0.306	
	*H_E_*	0.812	0.891	0.873	0.894	0.902	0.896	0.281	
San Miguel	*N*	26	32	26	32	32	31	34	30.5
	*A*	10	13	10	12	14	15	5	11.1
	*H_O_*	0.893	0.875	0.808	0.875	0.938	0.968	0.382	
	*H_E_*	0.829	0.878	0.858	0.882	0.871	0.927	0.358	
St. George	*N*	96	96	92	95	96	95	96	95.1
	*A*	13	16	12	14	18	16	10	14.0
	*H_O_*	0.865	0.833	0.880	0.895	0.906	0.937	0.333	
	*H_E_*	0.847	0.881	0.865	0.899	0.915	0.907	0.342	
St. Paul	*N*	104	106	97	103	106	104	106	104.0
	*A*	14	16	12	15	20	14	10	14.1
	*H_O_*	0.827	0.830	0.866	0.816	0.868	0.885	0.330	
	*H_E_*	0.821	0.876	0.858	0.879	0.900	0.894	0.365	
Mean all pop.s	*H_O_*	0.835	0.892	0.836	0.866	0.899	0.914	0.293	58.0
	*H_E_*	0.830	0.883	0.857	0.888	0.896	0.902	0.323	12.2
Total all pop.s	*N*	455	477	442	455	477	461	472	
	*A*	19	19	13	17	22	17	12	

*Sample size (*N*), number of alleles (*A*), observed (*H_O_*) and expected heterozygosity (*H_E_*) for each population and locus.

Probability tests for genotypic linkage disequilibrium for all pairs of loci within each population indicated nonrandom associations in one of 168 comparisons (St. George Island, Hg4.2 & Hg8.10, P = 0.0033; initial α = 0.05/8 = 0.0063). Due to small sample sizes, no information was given for 15 of 21 comparisons in the Lovushki Island population. No significant values were observed, however, for any of the locus-locus combinations across all populations (all *P*≥0.241).

**Figure 2:**
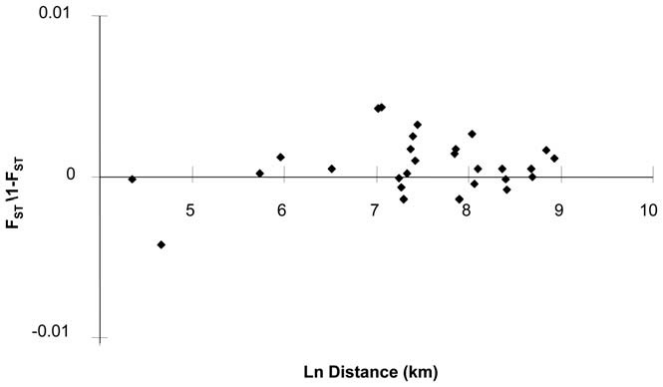
Isolation by distance based on microsatellites in northern fur seals including the relationship between genetic distance, pairwise comparisons of rookeries and the natural log of the geographic distance between the rookery pairs.

**Figure 3:**
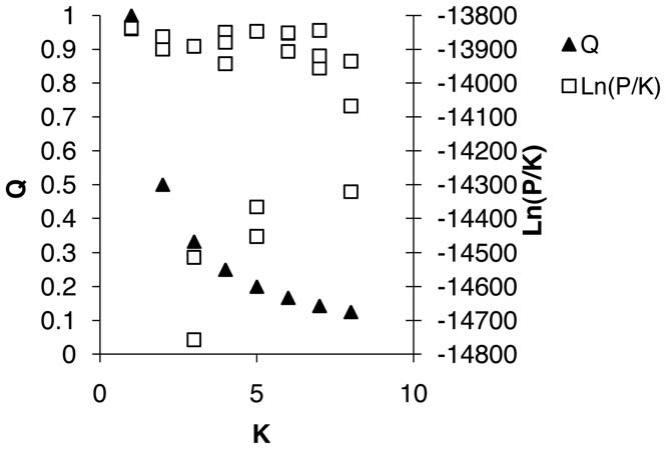
Likelihood (Ln[Pr(X/K)]) and mean maximum Q (proportion of ancestry for each individual assigned to a cluster) plots for STRUCTURE analysis using three runs each for K = 1 to 8.

Population differentiation at microsatellite loci, estimated by F_ST_ over all populations and loci, was not significant (*F_ST_* = 0.0004, *P* = 0.273). F_ST_ estimates over all populations by locus were also small and not significant (*F_ST_* ≤0.0026, *P*≥0.110). Multilocus estimates of F_ST_ for pairs of populations ranged from −0.0042 to 0.0043, and none were significant (*P*≥0.05). Mantel tests of isolation by distance for population pairs found no significant correlations between geographic distance and F_ST_/(1-F_ST_) (*P* = 0.410; Figure 2). There was also no significant difference in allelic richness between populations (N = 50, F = 0.013, df = 13, P = 0.91). Maximum likelihood tests examining the number of populations using an admixture model in STRUCTURE showed that the highest probabilities and lowest confidence intervals were found when all samples were grouped into one population (K = 1; Figure 3).

A total of 381 base pairs of the mtDNA control region (D-loop) were analyzed for sequence variation in 619 northern fur seals sampled throughout their range (Table 2). Eighty-seven variable sites were found with 106 substitutions (83 transitions and 23 transversions) and one indel. In total, 332 different haplotypes were identified, 227 of which were represented by single individuals (Table 2). Haplotypic diversity was high (h  = 0.994, SD = 0.0009) due to the large number of unique haplotypes, but nucleotide diversity was moderate (π = 2.4%, SD = 1.2%) suggesting that most haplotypes are closely related (Table 2).

**Table 2:** Summary of mtDNA diversity in 9 populations of Northern fur seal.

Location	Sample Size	Number of Haplotypes	Haplotypic Diversity, h	Nucleotide Diversity, Π
Robben Island	48	43	0.996 (0.006)	2.6 (1.3)
Lovushki Island	61	55	0.997 (0.004)	2.3 (1.2)
Srednev Island	49	45	0.994 (0.007)	2.4 (1.3)
Bering Island	48	41	0.993 (0.006)	2.4 (1.2)
Medney Island	48	43	0.996 (0.005)	2.4 (1.2)
Saint Paul Island	91	68	0.993 (0.003)	2.2 (1.1)
Saint George Island	92	76	0.992 (0.004)	2.4 (1.2)
Bogoslof Island	96	71	0.99 (0.004)	2.3 (1.2)
San Miguel Island	86	68	0.992 (0.004)	2.4 (1.2)
Total	619	332	0.994 (0.001)	2.4 (1.2)

*haplotypic diversity (h), % nucleotide diversity (Π). Standard deviations in parentheses.

**Figure 4:**
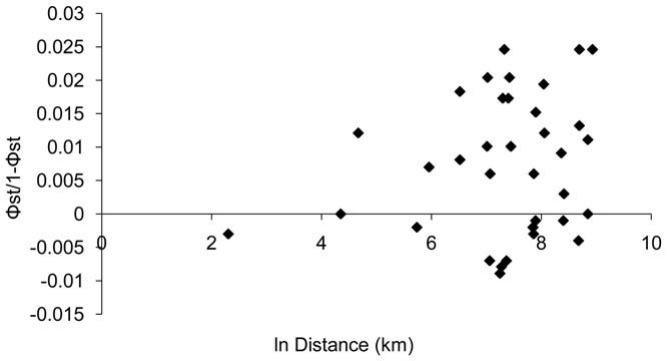
Isolation by distance based on mitochondrial DNA analysis in northern fur seals including the relationship between genetic distance, pairwise comparisons of rookeries (Φ_ST_ ) and the natural log of the geographic distance between the rookery pairs.

Overall, population differentiation using mtDNA was not significant among the six regional groupings: Robben Island; the Kuril Islands (Lovushki and Srednev); the Commander Islands (Bering and Medny); Bogoslof Island; the Pribilof Islands (St. George and St. Paul); and San Miguel Island (AMOVA, P = 0.87), nor was it significant when comparing the western Pacific islands (Robben Island, Kuril Islands, and Commander Islands) to the eastern Pacific islands (Pribilof Islands; AMOVA, P = 0.80). However, there was significant differentiation between some population pairs. Estimates of conventional F_ST_ values (based only on haplotype frequencies) were very low but significant for only 2 of 36 comparisons (Table 3). Φ_ST_ estimates (based on both haplotype frequencies and a measure of genetic distance) showed higher levels of differentiation among population pairs and statistical significance for 9 of 36 comparisons (Table 3). The majority of the differences were detected between U.S. islands and either Robben Island or Bering Island, suggesting some level of population structure between the western and eastern North Pacific Ocean. Although an analysis of isolation by distance was not significant (F = 1.24, df = 34, P = 0.27, r^2^ = 0.035; Figure 4).

**Table 3:** Mitochondrial DNA based population differentiation for population pairs (estimates of F_ST_ above diagonal and Φ_ST_ below diagonal).

	Robben	Lovushki	Sredengo	Bering	Medney	Bogoslof	St Paul	St George	San Miguel
Robben		−0.001	−0.001	−0.002	−0.001	−0.003	0.000	−0.003	0.000
Lovushki	0.013		0.000	0.001	0.001	0.001	0.002	0.002	0.001
Sredengo	**0.018**	−0.003		0.002	0.002	0.002	**0.004**	0.000	0.002
Bering	0.017	0.010	0.02		0.000	−0.002	0.000	−0.001	0.002
Medney	0.010	−0.007	0.006	0.012		−0.001	0.002	0.000	0.002
Bogoslof	0.010	−0.001	**0.015**	**0.02**	−0.007		0.002	−0.001	0.002
St Paul	**0.019**	−0.002	−0.002	**0.017**	−0.001	0.007		0.003	**0.005**
St George	**0.012**	−0.003	0.006	**0.025**	−0.008	−0.002	0.000		0.000
San Miguel	**0.024**	−0.000	0.011	**0.024**	−0.004	0.009	0.003	−0.001	

*Bold indicates significant *P*-values (*P*<0.05).

**Figure 5:**
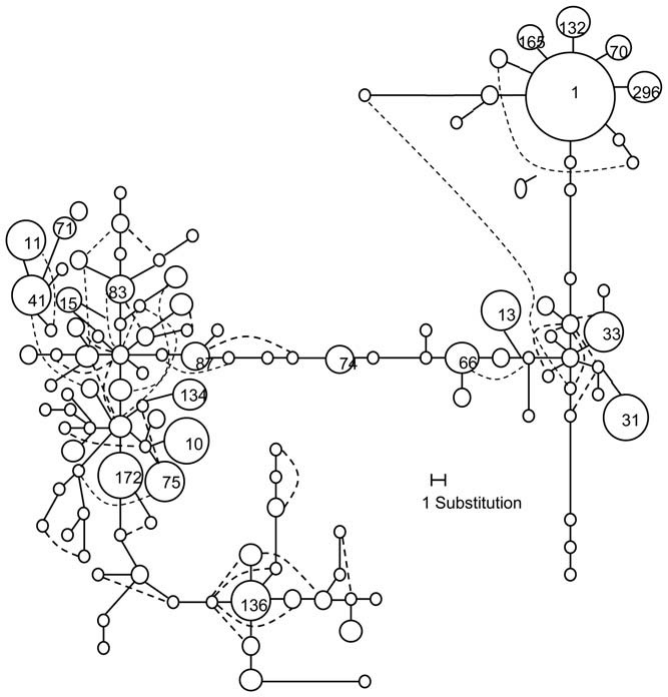
Minimum spanning network of 112 core mitochondrial DNA haplotypes of northern fur seals. Branch lengths are the minimum number of steps between haplotypes. The size of the circle representing the individual haplotypes corresponds to the abundance of that haplotype. Numbers identify the most abundant haplotypes. Dashed lines represent alternative groupings.

The minimum-spanning network shows three distinct maternal lineages generally characterized by a star-like pattern with long branches linking groups of more closely related haplotypes together (all terminal branches of the minimum spanning network were pruned to facilitate interpretation reducing the network to 112 core haplotypes; Figure 5). There does not appear to be a geographic basis for the lineages as representatives of all population groups were found in all three lineages.

**Figure 6:**
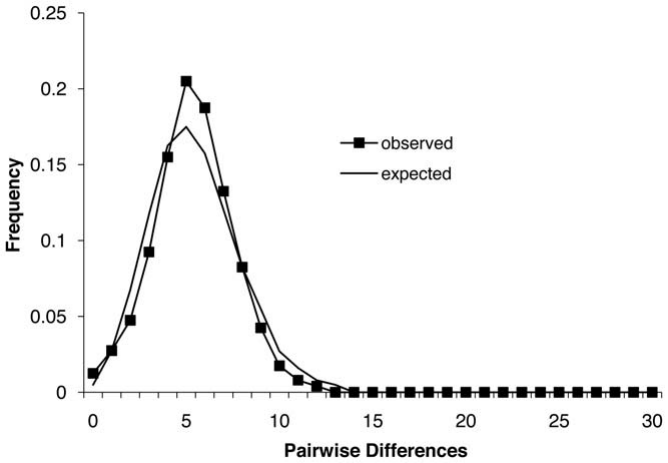
The observed pairwise mismatch distribution of mtDNA in northern fur seals as compared to the expected distribution based upon a model of sudden population expansion.

**Figure 7:**
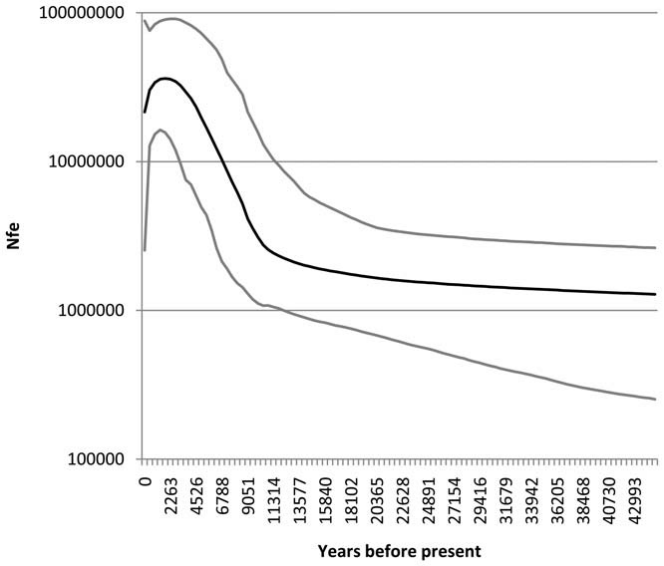
Bayesian skyline plot of historical female effective population size, light lines represent the 95% highest posterior probability density around the estimate.

Evidence for past population expansion was strong. The unimodality of the nucleotide frequency mismatch distribution was almost identical to a model of sudden expansion. These results suggest that northern fur seals have undergone a rapid expansion event in recent evolutionary history and that the signature of this event is still evident in their genetic composition (Figure 6). The results of the skyline analysis further support this conclusion showing a rapid increase in population size starting ∼11000 ybp, followed by a more recent decrease in population numbers starting ∼2000 ybp (Figure 7).

## Discussion

We found no evidence of population differentiation in the northern fur seal across seven microsatellite markers. Further, maximum likelihood tests suggest that animals distributed throughout the entire range form a single population and estimates of F_ST_ among island regions through this area were not significantly greater than zero. In addition, we found no evidence of an isolation by distance pattern among any of the sampled breeding islands. Admittedly, the number of loci used in this analysis is relatively low and may not confer sufficient power to discern fine-scale structure. The use of additional loci could help to elucidate patterns of genetic structure not identified in this study. This has been shown to be the case in Steller sea lions, an initial analysis using 6 microsatellite loci revealed no population structure [15] yet an increase in sample size and the use of 13 highly polymorphic microsatellites revealed genetic structure [16]. In contrast to the microsatellite results, pairwise comparisons of mtDNA Φ_ST_ estimates suggest low levels of differentiation between the Russian populations and those found in the east Pacific (Table 3). Male-mediated gene flow through sex-biased dispersal patterns or alternative mating tactics (e.g. Antarctic fur seal) [51], [52] could reduce the degree of population differentiation seen in microsatellites relative to maternally inherited mtDNA. Our results suggest that male biased dispersal may be contributing to the lack of genetic structure, however, the relatively low level of differentiation found in mtDNA suggests that this is not the explanation for the lack of genetic structure we characterized in the microsatellite analysis. Alternative mating tactics would likely result in mating events occurring at locations other than the rookery of interest [52] and could result in a reduction of genetic differentiation [53].

Our results suggest that migration has greatly influenced the genetic structure of northern fur seal breeding aggregations, probably both historically (through population expansion since the last glacial maximum) and more recently (through post-harvest expansions and contemporary migrations). Evidence of these events is clear in the star-like shape of the minimum spanning network, the pairwise mismatch distribution, and the skyline plot. First, the existence of a star phylogeny consisting of three distinct, but closely related, lineages with no relationship to geography (Figure 5) demonstrates rapid expansion in the evolutionarily recent past [54], and is could be the result of recolonization by animals from throughout the range. Additionally, we found an extremely close fit between the observed pairwise mismatch distribution and the expected distribution based on a model of rapid population expansion [45] (Figure 6). Finally, the skyline plot shows a period of rapid increase in population numbers following the last glacial retreat (∼11000 ypb) and a more recent (∼2000 ybp) reduction in population size. This more recent decline is worth further comment as it suggests that hunting pressure from early North American human cultures may have had an impact on the population (Figure 7). Interestingly, the upper confidence limit indicates a very recent increase in population size. Although the scale of the analysis does not allow us to examine this result further, it suggests a historically recent increase in population size following the relatively recent cessation of unregulated northern fur seal harvests.

Taken together our results demonstrate that the impacts of past population expansion in northern fur seals are still evident. While we cannot specifically identify which of the two examined processes, modern gene flow and historical recolonization, played the most important role in generating current genetic structure, we do know that rapid colonization does occur in northern fur seals. This is particularly evident in the recent, and rapid, colonization of Bogoslof (1980) [7], [8] and San Miguel (1965) [9] islands, both of which, based on tag-resight data, were colonized by animals from throughout the northern fur seal range. High rates of migration as a result of recolonization following perturbations in the population mixed with even small amounts of contemporary gene flow could have lead to genetic homogenization. Our results are similar to those found in other of otariid species that have undergone similar population perturbations [20], [21].

### Conclusion

Although northern fur seals appear to exhibit a high degree of behavioral philopatry in both sexes [2], [5], [6], have extensive geographic separation of breeding islands (Figure 1), marked differences in foraging behavior and habitat use around those islands [10], [55], [56], and differences in population dynamics [2], we found only weak genetic structure across their vast North Pacific range. The results of our study suggest that this lack of genetic structure results from a combination of insufficient time since rapid recolonization, during both post-glacial and post-harvest expansion, and contemporary migration between breeding colonies. Our findings demonstrate the importance of understanding temporal influences when characterizing population genetic structure. Specifically, the genetic influences of population processes can persist well beyond the abeyance of the processes themselves [57]. Thus, our study emphasizes the importance of investigating more than patterns of neutral genetic differentiation in attempts to characterize ecologically distinct populations [58].
